# Otolin-1 for differentiating central and peripheral causes of acute continuous dizziness

**DOI:** 10.1371/journal.pone.0361036

**Published:** 2026-10-22

**Authors:** Vincent W. Klokman, Merel J. J. Verhagen, Adriaan A. J. Ros, Karlijn F. de Laat, Korné Jellema, Dominique P. V. de Kleijn, Kim E. Jie

**Affiliations:** 1 Department of Emergency Medicine, Jeroen Bosch Hospital, ‘s-Hertogenbosch, the Netherlands; 2 Department of Vascular Surgery, University Medical Center Utrecht, Utrecht, the Netherlands; 3 Department of Neurology, Haga Hospital, The Hague, the Netherlands; 4 Department of Neurology, Maasstad Hospital, Rotterdam, the Netherlands; 5 Department of Neurology, St. Antonius Hospital, Nieuwegein, the Netherlands; 6 Department of Neurology, Haaglanden Medical Center, The Hague, the Netherlands; LSU Health Shreveport, UNITED STATES OF AMERICA

## Abstract

**Background:**

Patients with isolated acute-onset continuous dizziness (iAOCD) are commonly seen at the emergency department (ED). Distinguishing peripheral vestibular disorders from central causes remains challenging. Existing diagnostic tools, including bedside examination and neuroimaging, have important limitations. Otolin-1, an inner ear–specific glycoprotein, has been proposed as a biomarker of peripheral vestibular pathology. This study evaluated whether otolin-1 could serve as a diagnostic biomarker for this differentiation.

**Methods:**

This study analyzed samples from a prospective multicenter cohort of adult patients who were seen at the ED with acute onset, continuous dizziness without focal neurological deficits. Patients were included within 24 hours of symptom onset. Otolin-1 plasma concentrations were measured using an enzyme-linked immunosorbent assay. Final diagnoses (central versus peripheral) were determined by an expert panel based on clinical assessment, diffusion-weighted magnetic resonance imaging and 3-month follow-up.

**Results:**

We analyzed 245 patients (179 peripheral, 66 central). Median otolin-1 concentrations did not differ significantly between peripheral and central causes (517.6 [IQR 443.7–592.8] pg/mL vs 487.9 [424.9–644.4] pg/mL; p = 0.349). No significant differences were observed in hyperacute (<4.5 hours after symptom onset) or acute (4.5–24 hours after symptom onset) subgroups. Similarly, otolin-1 concentrations did not differ between central iAOCD and specific peripheral diagnoses. Substantial overlap in concentration ranges was observed across all comparisons. Receiver operating characteristic analysis similarly showed no useful discrimination (AUC 0.539, 95% CI 0.455–0.623).

**Conclusions:**

Otolin-1 does not differentiate between central and peripheral causes of iAOCD in the ED setting. Although biologically linked to inner ear pathology, otolin-1 lacks sufficient discriminatory value as a stand-alone diagnostic biomarker.

## Introduction

Acute dizziness or vertigo is a common reason for presentation to the emergency department (ED) [1,2]. In patients with acute vestibular syndrome (AVS), the underlying cause is most often a peripheral vestibular disorder, such as vestibular neuritis or labyrinthitis. However, approximately 20% of patients have central causes, particularly posterior circulation stroke [3]. Acute vestibular syndrome (AVS) is a term used to describe acute-onset, severe dizziness, imbalance or vertigo lasting more than 24 hours, with exam findings of nystagmus or gait unsteadiness [1]. However, not all patients with acute dizziness present with a visible or spontaneous nystagmus, and patients in the ED are often evaluated within the first hours after symptom onset, before this duration and nystagmus criterion can be fulfilled. For this reason, we use the term isolated acute-onset continuous dizziness (iAOCD) to describe patients presenting with acute, ongoing dizziness without focal neurological deficits, representing a clinically relevant umbrella population that includes patients with evolving AVS. In this setting, early differentiation between peripheral vestibular disorders and central causes, particularly posterior circulation ischemia, remains challenging.

Bedside examination techniques, specifically the Head-Impulse test, Nystagmus, Test-of-Skew and hearing (HINTS+) examination, have demonstrated high diagnostic accuracy when performed by experienced clinicians [4]. However, their performance is reduced when applied by non-specialized physicians [5]. Neuroimaging also has recognized limitations. Non-contrast computed tomography (CT) has low sensitivity for acute ischemic stroke [6], while diffusion-weighted magnetic resonance imaging may fail to detect up to 15% – 20% of posterior circulation infarctions within the first 24–48 hours, particularly in small lesions [7–9].

These limitations have prompted interest in circulating biomarkers as adjunctive tools for differentiating central from peripheral causes of iAOCD in the acute setting. However, current evidence remains insufficient to support their routine clinical use [10]. Biomarkers that are specific to inner ear pathology may be particularly informative, as they could provide objective evidence supporting a peripheral vestibular origin and thereby reduce the clinical likelihood of a central cause. Among these, the inner ear–specific protein otolin-1 has emerged as a promising candidate with potential diagnostic utility [11].

Otolin-1 is a secreted glycoprotein expressed in the supporting structures of the cochlear and vestibular sensory organs, where it contributes to the structural organization of the otoconial matrix [12,13]. Given its expression is mainly in otolithic structures, the presence of otolin-1 in peripheral blood has been proposed as a marker of inner ear injury [14]. Circulating otolin-1 levels have been shown to be increased in patients with benign paroxysmal positional vertigo (BPPV), consistent with otoconia degeneration and displacement [14–17]. Elevated otolin-1 levels have also been reported in other peripheral vestibular disorders, including Ménière’s disease [18,19] and acute unilateral vestibulopathy [18,20], suggesting that otolin-1 reflects inner ear injury rather than a disease-specific process.

While this supports its role as a sensitive biomarker of peripheral vestibular pathology, it also limits its specificity for individual disease entities. The potential role of otolin-1 in differentiating central from peripheral causes of acute dizziness has not been investigated. Given that central causes do not directly involve otoconial damage, it is plausible that circulating otolin-1 concentrations differ between these groups. Conversely, if otolin-1 is a marker of chronic turnover rather than acute injury, no difference would be expected. The objective of the present study was therefore to evaluate the diagnostic utility of otolin-1 in distinguishing central from peripheral causes of iAOCD in patients presenting to the ED.

## Materials and methods

### Ethical approval

This study was conducted in accordance with the principles of the Declaration of Helsinki. Ethical approval was obtained from the Medical Ethics Committee Leiden The Hague Delft of the Dutch Central Committee on Research Involving Human Subjects (protocol number NL76143.058.21; registration number P21.029). Written informed consent was obtained from all participants.

### Study design and population

This study was conducted using blood samples from patients who were included in the PROVIDE study (Providing Diagnostic Accuracy in Acute Onset, Continuous Dizziness), a prospective multicenter cohort study. The PROVIDE study enrolled adult patients who were seen at the ED with acute onset, continuous dizziness in the absence of focal neurological deficits.

Between August 1, 2021, and July 1, 2024, a total of 800 patients were included across 11 hospitals in the Netherlands (2 academic and 9 non-academic teaching hospitals). Blood samples were collected from 645 patients. The primary endpoint of the PROVIDE study was the final diagnosis established after 3 months of follow-up.

For the present analysis, 245 subjects were selected from the PROVIDE cohort using a stratified sampling approach based on diagnostic group. To ensure adequate representation of central causes, 66 patients with a central diagnosis and available neuroimaging were selected first. Thereafter, patients with a peripheral diagnosis, including vestibular neuritis, BPPV or Ménière’s disease, were randomly selected from the remaining eligible PROVIDE samples. A priori power calculations, based on a pooled effect size from a prior meta-analysis (standardized mean difference: 1.53) [11], indicated that at least 128 patients with peripheral iAOCD and 64 patients with central iAOCD, using a 2:1 ratio, would provide more than 90% power (α = 0.05) to detect a clinically relevant medium effect size (Cohen’s d = 0.5) in otolin-1 plasma concentrations. Fig 1 depicts the study in a STARD flow diagram.

**Fig 1:**
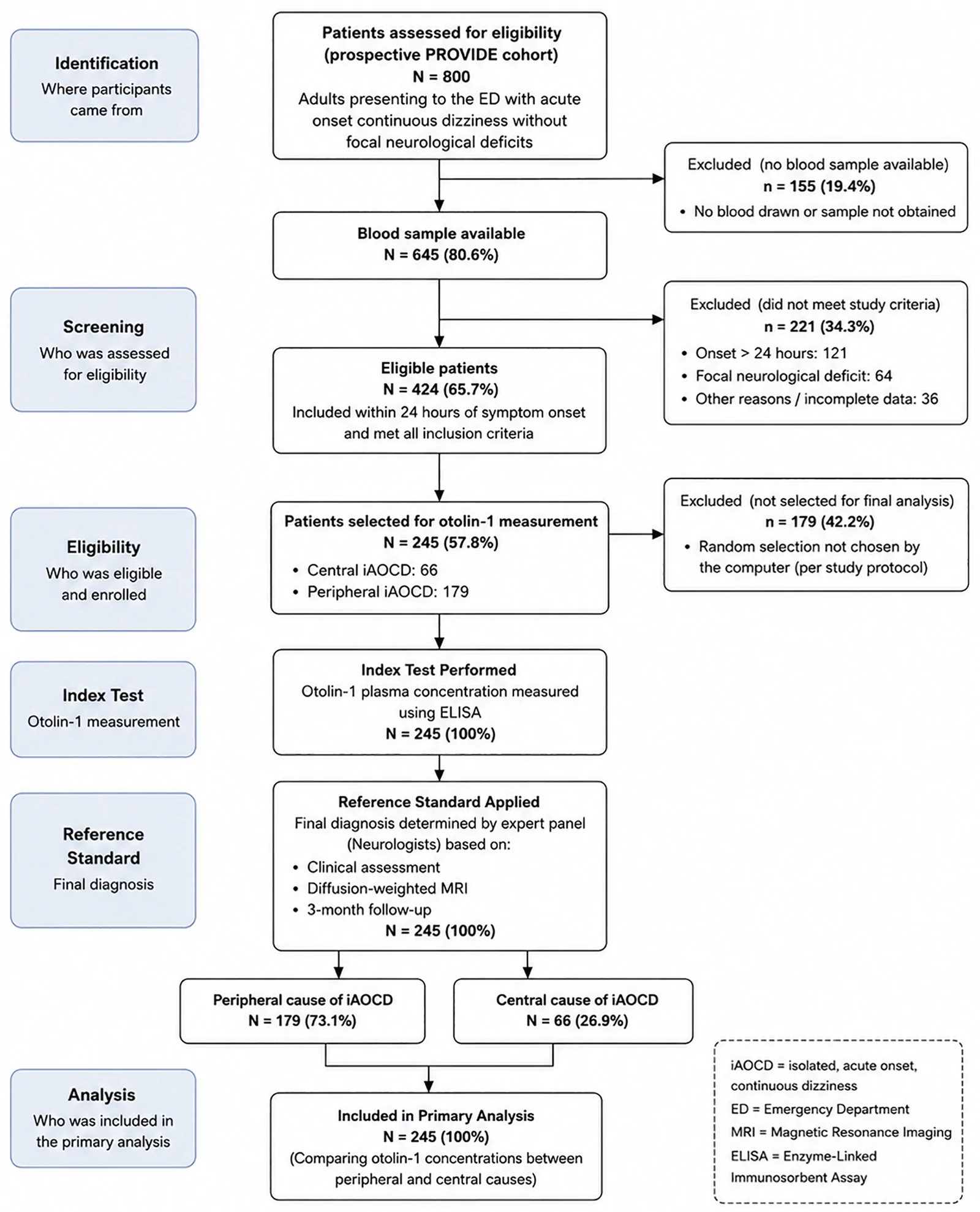
STARD flow diagram.

### Inclusion and exclusion criteria

Patients were eligible for inclusion if they met the following criteria: (1) acute onset continuous dizziness still present at ED arrival as the primary complaint; (2) presentation within 24 hours of symptom onset; and (3) age ≥ 18 years.

Exclusion criteria were: (1) recurrent peripheral AVS or symptoms compatible with a previously established diagnosis (e.g., Ménière’s disease or vestibular migraine); (2) new focal neurological deficits on examination (e.g., ataxia, dysarthria, spontaneous skew deviation, gaze palsy, or reduced level of consciousness defined as Glasgow Coma Scale less than 14); (3) Clear signs of BPPV (i.e., iAOCD successfully treated by canalith repositioning) (4) known pregnancy; (5) contraindications to magnetic resonance imaging (MRI), such as claustrophobia or non-compatible implanted devices; (6) a clear alternative medical condition explaining symptoms (e.g., hypotension, sepsis, or medication-related causes); (7) prior inclusion in the PROVIDE study; (8) inability to complete follow-up (e.g., life expectancy less than 3 months, severe cognitive impairment, or lack of permanent residence in the Netherlands); (9) inability to provide informed consent; or (10) insufficient command of the Dutch language. The presence of nystagmus and gait imbalance or instability was permitted but not required.

### Clinical data and outcome assessment

Baseline data collected at presentation included demographic characteristics, medical history, and symptom characteristics.

The final diagnosis (peripheral vestibular disorder vs central cause) was established by an expert panel consisting of experienced neurologists in this field. The diagnosis was based on clinical findings, including HINTS (plus) examination results, diffusion-weighted MRI and outpatient clinical follow-up at 3 months. The expert panel were blinded to the otolin-1 concentration results.

### Sample collection and biochemical analysis

Blood samples were collected in 4 mL K3-EDTA tubes at ED presentation. Samples were centrifuged at 2500 × g for 10 minutes, and the supernatant was aliquoted and stored at −80°C until analysis. Otolin-1 concentrations were measured using a commercially available enzyme-linked immunosorbent assay (ELISA) kit (catalog number MBS167289; MyBiosource, San Diego, CA, USA), based on a biotin double-antibody sandwich technique. All assays were performed in accordance with the manufacturer’s instructions. General laboratory values were carried out by each participating institution per local protocol. Laboratory personnel were blinded to clinical data and final diagnoses during sample analysis.

### Statistical analysis

Statistical analyses were performed using R (version 4.4.0). Continuous variables are presented as mean ± standard deviation (SD) for normally distributed data, or as median with interquartile range [IQR] for non-normally distributed data. Categorical variables are presented as counts and percentages. Normality of continuous variables was assessed using the Shapiro–Wilk test. Group comparisons were performed using the independent samples t-test for normally distributed data and the Wilcoxon rank-sum test (Mann–Whitney U test) for non-normally distributed data. Baseline characteristics were summarized descriptively using available observations; missing data were not imputed. No formal statistical testing was performed for baseline characteristics, as the diagnostic groups were not randomized and were expected to differ clinically. A p-value <0.05 was considered statistically significant.

As a secondary analysis, receiver operating characteristic (ROC) curves were constructed to assess the ability of plasma otolin-1 concentrations to discriminate peripheral from central iAOCD. Peripheral etiology was defined as the positive outcome, consistent with the hypothesis that higher otolin-1 concentrations indicate peripheral vestibular pathology. Areas under the ROC curve (AUCs) were calculated with 95% confidence intervals. ROC analyses were performed for the overall cohort and separately for the hyperacute (0–4.5 hours) and acute (4.5–24 hours) subgroups.

## Results

### Study population

Following the stratified selection procedure, the final study population consisted of 245 patients: 66 patients with central iAOCD and 179 randomly selected patients with peripheral iAOCD. Baseline characteristics are shown in Table 1. Patients with central iAOCD more frequently had vascular risk factors, including hypertension, hypercholesterolemia and prior ischemic stroke, whereas age and sex distribution were comparable between groups. Median duration of symptoms at blood sampling was also comparable between patients with a peripheral or central cause (6.75 [3.08–13.50] vs 7.08 [3.19–15.31] hours).

**Table 1:** Clinical characteristics of subjects.

Characteristics	Peripheral iAOCD (n = 179)	Central iAOCD (n = 66)
Sex (%Male)	49.2	48.5
Age	70.0 (60-75)	70.0 (59.25-77)
Duration of Symptoms at time of sampling (hours)	6.75 [3.08,13.50]	7.08 [3.19,15.31]
Prior Medical History		
Hypertension	78 (43.6%)	41 (62.1%)
Diabetes mellitus	14 (7.9%)	11 (16.7%)
Hypercholesterolemia	55 (30.7%)	33 (50.0%)
Atrial Fibrillation	12 (6.7%)	4 (6.1%)
Obstructive Sleep Apnea	12 (6.7%)	5 (7.6%)
Migraine	12 (6.7%)	3 (4.5%)
Transient Ischemic Attacks	13 (7.3%)	8 (12.1%)
Ischemic stroke	14 (7.8%)	13 (19.7%)
Hemorrhagic stroke	0 (0.0%)	1 (1.5%)
Myocardial Infarction	16 (8.9%)	8 (12.1%)
Peripheral Arterial Vascular Disease	5 (2.8%)	2 (3.0%)
Hypercoagulability	1 (0.6%)	0 (0.0%)
Familial Cardiovascular Disease	50 (27.9%)	20 (30.3%)
Clinical and Lab Characteristics		
Systolic Blood Pressure (mmHg)	158 [143.00, 174.50]	163 [149.00, 175.00]
Diastolic Blood Pressure (mmHg)	85 [76.50, 93.00]	86.50 [77.00, 97.75]
Hemoglobin (mmol/L)	8.80 [8.38, 9.40]	8.70 [8.05, 9.25]
Hematocrit (%)	0.42 [0.40, 0.45]	0.42 [0.39, 0.44]
MCV (fL)	90.00 [88.00, 93.00]	90.00 [85.00, 93.00]
Leukocyte Counts (x10^9^/L)	8.15 [6.80, 10.00]	8.45 [7.00, 10.73]
C-reactive Protein (mg/L)	2.00 [1.00, 3.50]	2.80 [1.00, 5.00]
Glucose (mmol/L)	7.40 [6.30, 8.40]	8.15 [6.93, 9.75]
Sodium (mmol/L)	139.4 [136.9, 141.9]	138.7 [136.4, 141.0]
Potassium (mmol/L)	4.00 [3.60, 4.20]	3.80 [3.50, 4.20]
Creatinine (µmol/L)	73.00 [62.25, 85.00]	75.00 [61.50, 87.50]
Blood Urea Nitrogen (mmol/L)	5.80 [4.80, 6.80]	6.10 [4.78, 7.20]
Glomerular Filtration Rate- CKD-EPI (mL/min/1.73m^2^)	81.50 [69.00, 90.00]	81.00 [69.00, 90.00]
Low density Lipoprotein (mmol/L)	2.80 [2.20, 3.48]	3.25 [2.32, 3.88]
High density Lipoprotein (mmol/L)	1.40 [1.10, 1.60]	1.29 [1.10, 1.50]
Triglycerides (mmol/L)	1.40 [1.02, 1.77]	1.14 [1.00, 1.60]
Total Cholesterol (mmol/L)	4.80 [4.10, 5.60]	5.00 [4.00, 5.70]

Abbreviations: iAOCD, isolated acute-onset continuous dizziness. Values are presented as n (%) or median [interquartile range], unless otherwise indicated. Values are based on available observations. Missing data were not imputed; data is reported in S1 Dataset.

### Otolin-1 concentrations

Median otolin-1 concentrations did not differ significantly between patients with peripheral and central causes (517.6 [443.7–592.8] pg/mL vs 487.9 [424.9–644.4] pg/mL, p = 0.349; Table 2). Fig 2 shows the distribution of otolin-1 concentrations in peripheral and central iAOCD. Several peripheral cases had otolin-1 concentrations above the range observed in central cases; however, substantial overlap in concentrations remained between the two groups. Subgroup analyses regarding sampling time were carried out correlating to the thrombolysis window of less than 4.5 hours (i.e., hyperacute subgroup) and between 4.5 and 24 hours (i.e., acute subgroup). In patients presenting within 4.5 hours of symptom onset median otolin-1 concentrations were 522.1 pg/mL (IQR 443.7–592.8) in peripheral iAOCD and 535.3 pg/mL (451.2–668.5) in central iAOCD (p = 0.711). In patients who were seen between 4.5 and 24 hours after symptom onset, median concentrations were 508.1 pg/mL (444.1–592.7) and 484.4 pg/mL (420.3–554.0), respectively (p = 0.155) (Table 2). Figs 3 and 4 show the distribution and concentration ranges of otolin-1 in the hyperacute and acute onset subgroups, respectively.

**Table 2:** Comparison of otolin-1 concentrations between central iAOCD and peripheral diagnostic subgroups.

Characteristics	Peripheral Etiology	Central Etiology	p-value
Overall	517.6 pg/mL (n = 179)	487.9 pg/mL (n = 66)	0.349
Hyperacute subgroup (0–4.5 hours after symptom onset)	522.1 pg/mL (n = 67)	535.3 pg/mL (n = 23)	0.711
Acute subgroup (4.5–24 hours after symptom onset)	508.1 pg/mL (n = 112)	484.4 pg/mL (n = 43)	0.155
BPPV vs. Central iAOCD	506.6 pg/mL (n = 36)	487.9 pg/mL (n = 66)	0.713
Vestibular Neuritis vs. Central iAOCD	520.5 pg/mL (n = 131)	487.9 pg/mL (n = 66)	0.362
Ménière’s disease vs. Central iAOCD	522.0 pg/mL (n = 12)	487.9 pg/mL (n = 66)	0.387

Abbreviations: BPPV, Benign Paroxysmal Positional Vertigo; iAOCD, isolated acute-onset continuous dizziness.

**Fig 2:**
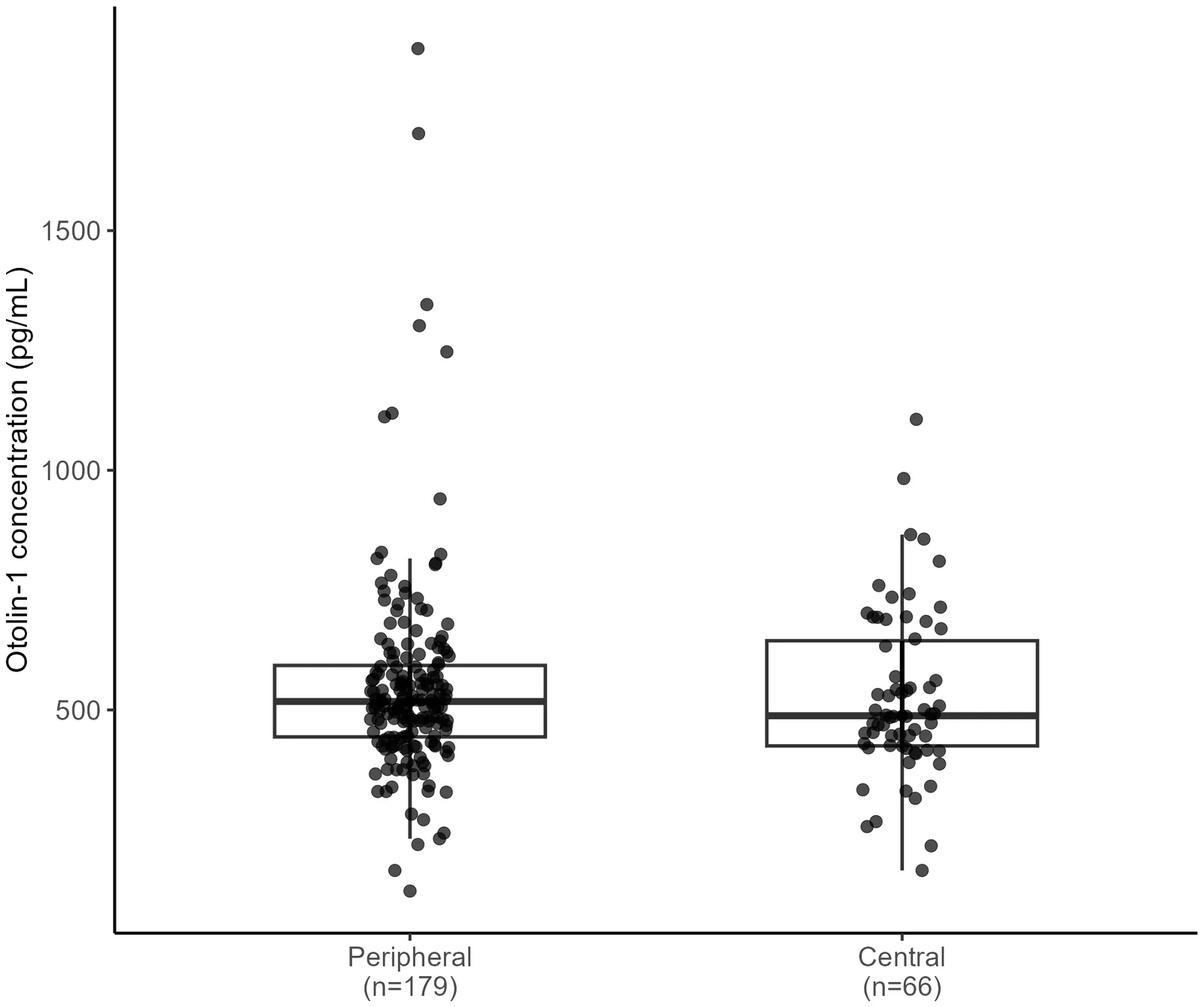
Otolin-1 concentration distributions in patients with peripheral and central isolated, acute onset, continuous dizziness.

**Fig 3:**
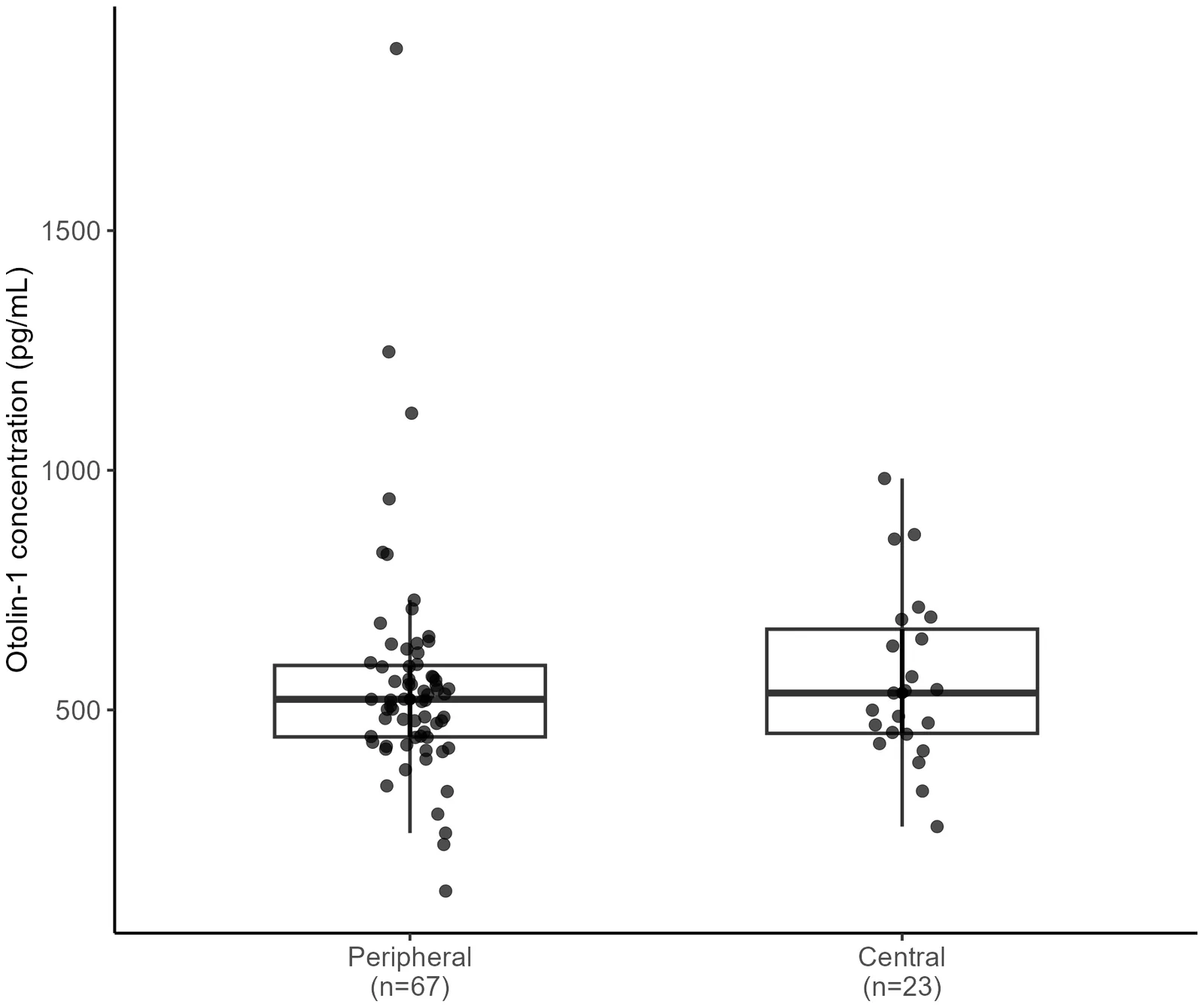
Otolin-1 concentration distributions in patients with peripheral and central isolated, acute onset, continuous dizziness within 4.5 hours of symptom onset.

**Fig 4:**
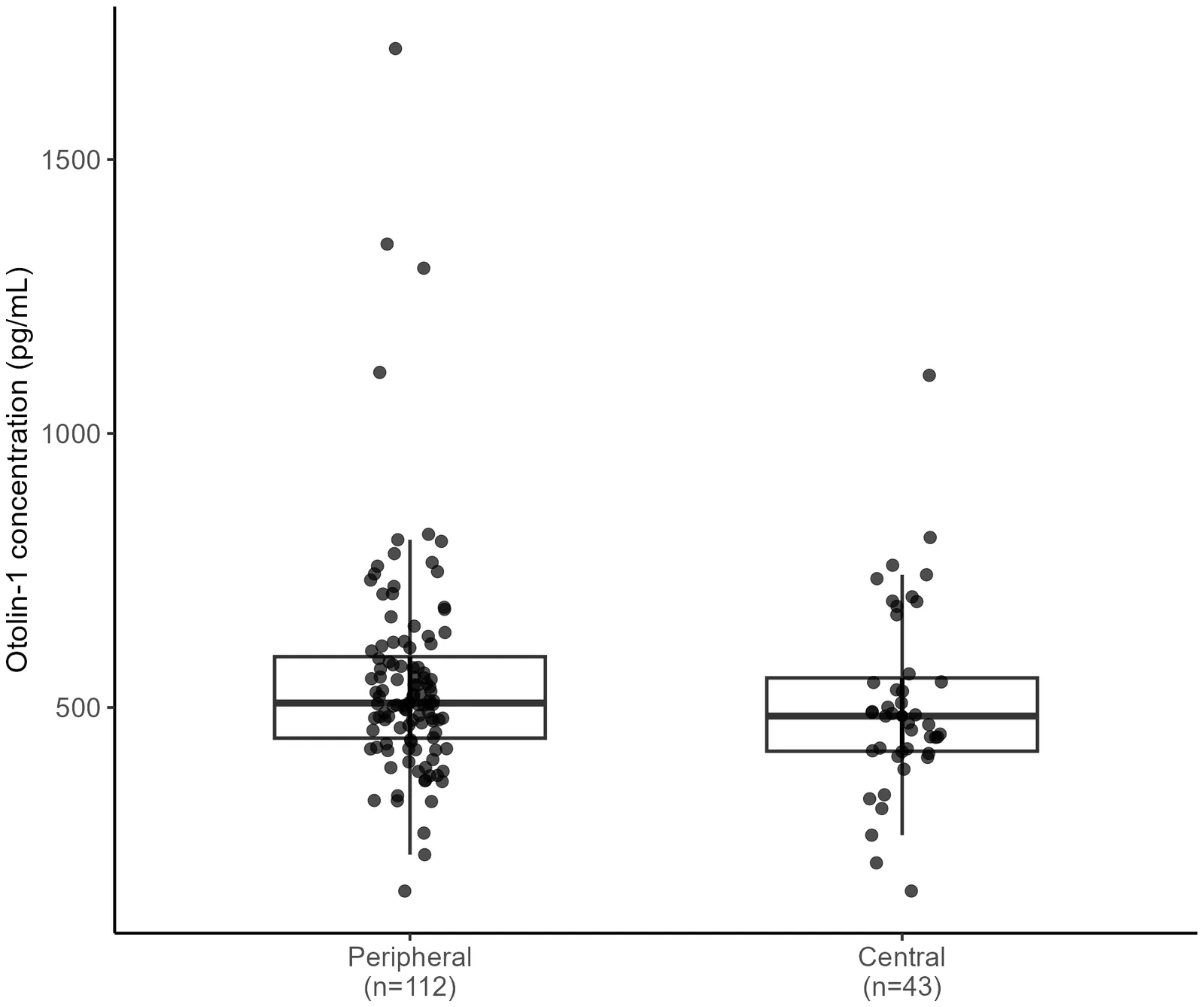
Otolin-1 concentration distributions in patients with peripheral and central isolated, acute onset, continuous dizziness between 4.5 and 24 hours after symptom onset.

Secondary ROC analysis confirmed poor discriminatory performance. In the overall cohort, the AUC was 0.539 (95% CI 0.455–0.623; S1 Fig). The AUC was 0.474 (95% CI 0.328–0.618) in the hyperacute subgroup sampled within 4.5 hours (S2 Fig) and 0.574 (95% CI 0.467–0.677) in the acute subgroup sampled between 4.5 and 24 hours after symptom onset (S3 Fig). All confidence intervals included 0.50.

### Otolin-1 concentrations in peripheral iAOCD subgroups

Median otolin-1 concentrations did not differ significantly between central iAOCD and individual peripheral vestibular diagnoses (Table 2). Fig 5 shows the distribution and concentration ranges of otolin-1 across central iAOCD and the peripheral diagnostic subgroups.

**Fig 5:**
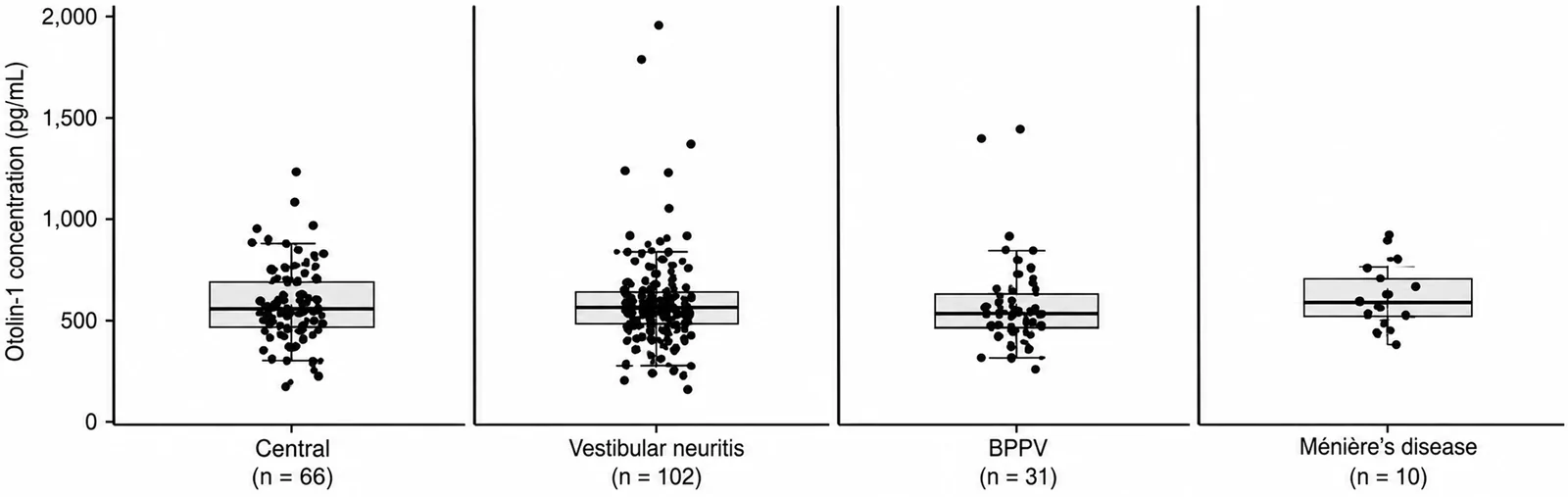
Otolin-1 concentration distributions in patients with central isolated, acute onset, continuous dizziness and the individual peripheral etiologies*. * From left to right: central isolated, acute onset, continuous dizziness, vestibular neuritis, BPPV, and Ménière’s disease.

## Discussion

In this prospective multicenter cohort, plasma otolin-1 concentrations did not differ significantly between patients with peripheral and central iAOCD, neither overall, stratified by symptom duration, nor within subgroups of specific peripheral vestibular diagnosis. These findings suggest that circulating otolin-1, despite its biological plausibility as a marker of peripheral vestibular pathology, does not provide discriminatory value for distinguishing peripheral from central causes of iAOCD in the emergency setting.

This study addresses a clinically relevant and previously unexplored question. Although otolin-1 has been proposed as a biomarker of peripheral vestibular pathology, particularly BPPV [14,15,17,21,22], to our knowledge no prior study has evaluated its ability to differentiate central from peripheral causes of dizziness.

Multiple studies have reported increased otolin-1 concentrations in BPPV compared with asymptomatic controls, and a recent meta-analysis demonstrated a pooled association between elevated otolin-1 levels and peripheral vestibular syndromes [11,21]. Similar findings have been reported in vestibular neuritis and Ménière’s disease, suggesting broader relevance of otolin-1 as a marker of peripheral vestibular injury [11,18]. However, those studies primarily addressed disease-versus-control discrimination rather than the more clinically demanding question of central versus peripheral differentiation. Our findings suggest that while otolin-1 may reflect otoconial turnover or inner ear pathology, this signal is not sufficiently disease-specific to distinguish peripheral from central iAOCD in acute clinical presentations.

Several biological explanations may account for these findings. One possibility is that otolin-1 reflects chronic otolithic degeneration or susceptibility rather than acute-state injury. Age-related increases in circulating otolin-1 have been described, potentially reflecting ongoing otoconia turnover independent of acute disease [23]. Additionally, peripheral vestibular disorders are biologically heterogeneous and do not uniformly involve direct injury to otolithic structures [11, 24]. While BPPV is closely related to otoconia degeneration and displacement, disorders such as vestibular neuritis primarily involve the vestibular nerve and may therefore not be expected to cause substantial otolin-1 release. Similarly, other peripheral vestibular disorders, such as semicircular canal dehiscence, can produce vestibular symptoms without primary otoconial injury. Consequently, grouping mechanistically distinct peripheral disorders may attenuate an otolin-1 signal that is present only in specific inner-ear pathologies. The substantial overlap in concentrations observed across diagnostic groups and peripheral subtypes is consistent with this possibility.

An important strength of this study is the rigor of the diagnostic reference standard against which this biomarker was tested. Unlike many prior biomarker studies relying on syndromic diagnoses or case-control comparisons, this cohort underwent robust phenotyping including MRI-driven diagnosis, expert panel adjudication, and three-month clinical follow-up, minimizing diagnostic misclassification. This is particularly relevant in AVS, where early neuroimaging may miss posterior circulation ischemia and diagnostic uncertainty remains substantial [4,7,8,25,26]. By using a stringent reference standard, these findings likely provide a more reliable estimate of the true discriminatory utility of otolin-1 than studies using less rigorous diagnostic classification.

These findings also have implications for biomarker development in acute iAOCD and AVS more broadly. Our results suggest that a single peripheral tissue–derived biomarker may be insufficient for this purpose. The absence of significant differences across symptom-duration strata is also noteworthy. If otolin-1 were released dynamically in response to acute peripheral injury, one might expect differences to be most apparent in hyperacute presentations. However, no such temporal signal was observed, arguing against a robust acute-release phenomenon detectable within the first 24 hours, although serial sampling studies are needed to define biomarker kinetics more precisely.

### Limitations

This study has several limitations. First, the sample size may still have limited power to detect modest effect sizes, particularly in subgroup analyses. However, the absence of even directional trends favoring discriminatory utility across analyses argues against a large clinically meaningful effect which might have been missed. Second, a single time-point biomarker measurement may not capture temporal kinetics of otolin-1 release. If otolin-1 behaves as a transient or delayed-response biomarker after acute peripheral vestibular injury, diagnostically relevant changes may have been missed. Nonetheless, the lack of signal even in hyperacute presentations argues against a strong early release pattern suitable for emergency diagnostic use. Third, although MRI-driven diagnosis, expert adjudication, and 3-month follow-up substantially reduce misclassification bias, some concern regarding occult MRI-negative posterior circulation stroke or transient ischemic attack is unavoidable in AVS research [7–9]. However, delayed follow-up and expert panel adjudication strengthen diagnostic certainty compared with many prior biomarker studies. Fourth, spectrum effects may have influenced results. Peripheral vestibular disorders such as vestibular neuritis, BPPV, and Ménière’s disease likely differ biologically, while central causes encompass heterogeneous stroke mechanisms and lesion sizes. It remains possible that biomarker signals exist in narrower phenotypic subsets not detectable in syndrome-level analyses.

Importantly, negative biomarker studies are informative in a field vulnerable to early optimism surrounding biologically plausible markers. Demonstrating lack of discriminatory performance is as important as demonstrating positive signal, because it helps focus future research toward more promising strategies.

## Conclusions

Otolin-1 concentrations were not significantly different between peripheral and central causes of iAOCD and did not support a role for otolin-1 as a stand-alone biomarker for differentiating these conditions. While otolin-1 remains of biological interest as a marker of peripheral vestibular pathology, these findings do not support its clinical use as a diagnostic discriminator in patients presenting with iAOCD or possible evolving AVS.

## Supporting information

S1 Fig Receiver operating characteristic curve for plasma otolin-1 in the overall cohort. ROC curve evaluating discrimination between peripheral and central iAOCD.
(TIFF)

S2 Fig Receiver operating characteristic curve for plasma otolin-1 in the hyperacute subgroup. ROC curve for patients sampled within 4.5 hours of symptom onset.
(TIFF)

S3 Fig Receiver operating characteristic curve for plasma otolin-1 in the acute subgroup. ROC curve for patients sampled between 4.5 and 24 hours after symptom onset.
(TIFF)

S1 Dataset De-identified participant-level dataset underlying the study findings. The workbook contains the data used for Tables 1 and 2, Figs 2–5, and the ROC analyses in S1–S3 Figs, together with a data dictionary and a summary of missingness.
(XLSX)
