# Supplementary material for: Otolin-1 for differentiating central and peripheral causes of acute continuous dizziness
Source: PLoS One. 2026 Oct 22;21(10):e0361036. doi: 10.1371/journal.pone.0361036 (PMC13690215; doi:10.1371/journal.pone.0361036)
Supplement: Supplementary file 1 [file pone.0361036.s005.docx]

**Response to Editors and Reviewers**

**Manuscript: “Otolin-1 for differentiating central and peripheral causes of acute continuous dizziness”**

We thank the Editor and both reviewers for their careful assessment and constructive comments. We have revised the manuscript accordingly. Changes are shown in the tracked-changes version and line numbers correspond accordingly.

# Editor comments

1. Please ensure that your manuscript meets PLOS ONE's style requirements, including those for file naming.

- We revised the manuscript and submission package to conform to PLOS ONE style, including manuscript formatting, figure and Supporting Information nomenclature, reference formatting, and file naming. A clean manuscript and a tracked-changes manuscript are being provided separately, and figure/Supporting Information files are named to match their in-text designations.

2. We note that the grant information you provided in the ‘Funding Information’ and ‘Financial Disclosure’ sections do not match.

- We reviewed the funding information and will ensure that the Funding Information and Financial Disclosure fields in the online submission system contain identical and correct information. The ZonMw award number is 10330022010010; the remaining named funders are listed without grant numbers where no specific award number applies.

3. Please state what role the funders took in the study.  If the funders had no role, please state: "The funders had no role in study design, data collection and analysis, decision to publish, or preparation of the manuscript." 
If this statement is not correct you must amend it as needed. 
Please include this amended Role of Funder statement in your cover letter; we will change the online submission form on your behalf.

- We have added the requested statement to the cover letter and will ensure that the online Funding Statement reflects it.

4. Please note that funding information should not appear in any section or other areas of your manuscript. We will only publish funding information present in the Funding Statement section of the online submission form. Please remove any funding-related text from the manuscript.

- The Funding section and all funding-related text have been removed from the manuscript.

Relevant manuscript line(s): Line 375 (deleted in the tracked-changes manuscript).

5. When completing the data availability statement of the submission form, you indicated that you will make your data available on acceptance. We strongly recommend all authors decide on a data sharing plan before acceptance, as the process can be lengthy and hold up publication timelines. Please note that, though access restrictions are acceptable now, your entire data will need to be made freely accessible if your manuscript is accepted for publication. This policy applies to all data except where public deposition would breach compliance with the protocol approved by your research ethics board. If you are unable to adhere to our open data policy, please kindly revise your statement to explain your reasoning and we will seek the editor's input on an exemption. Please be assured that, once you have provided your new statement, the assessment of your exemption will not hold up the peer review process.

- We have completed the data-sharing process. The de-identified participant-level dataset underlying the study findings has been added under Supporting Information as S4 Dataset.

Updated request from 3-10-2026

We've checked your submission and before we can proceed, we need you to address the following issues:

 We note that there is identifying data in the Supporting Information file S4 Dataset.xlsx. Due to the inclusion of these potentially identifying data, we have removed this file from your file inventory. Prior to sharing human research participant data, authors should consult with an ethics committee to ensure data are shared in accordance with participant consent and all applicable local laws.

Data sharing should never compromise participant privacy. It is therefore not appropriate to publicly share personally identifiable data on human research participants. The following are examples of data that should not be shared:

- Name, initials, physical address
- Ages more specific than whole numbers
- Internet protocol (IP) address
- Specific dates (birth dates, death dates, examination dates, etc.)
- Contact information such as phone number or email address
- Location data
- ID numbers that seem specific (long numbers, include initials, titled “Hospital ID”) rather than random (small numbers in numerical order)

Data that are not directly identifying may also be inappropriate to share, as in combination they can become identifying. For example, data collected from a small group of participants, vulnerable populations, or private groups should not be shared if they involve indirect identifiers (such as sex, ethnicity, location, etc.) that may risk the identification of study participants.

Additional guidance on preparing raw data for publication can be found in our Data Policy (<https://journals.plos.org/plosone/s/data-availability#loc-human-research-participant-data-and-other-sensitive-data>) and in the following article: <http://www.bmj.com/content/340/bmj.c181.long>.

Please remove or anonymize all personal information (<specific identifying information in file to be removed>), ensure that the data shared are in accordance with participant consent, and re-upload a fully anonymized data set. Please note that spreadsheet columns with personal information must be removed and not hidden as all hidden columns will appear in the published file.

We've returned your manuscript to your account. Please resolve these issues and resubmit your manuscript within 21 days. If you need more time, please email the journal office at [plosone@plos.org](mailto:plosone@plos.org). We are happy to grant extensions of up to one month past this due date. If we do not hear from you within 21 days, we will withdraw your manuscript.

Please log on to PLOS Editorial Manager at <https://www.editorialmanager.com/pone/> to access your manuscript. You will find your manuscript in the 'Submissions Sent Back to Author' link under the New Submissions menu. Be sure to remove your previous manuscript file if you are uploading a new file in response to these requests. After you've made the changes requested above, please be sure to view and approve the revised PDF after rebuilding the PDF to complete the resubmission process.

We are requesting these changes to comply with the PLOS One submission guidelines (<https://journals.plos.org/plosone/s/submission-guidelines>). Please note that we won't send your manuscript for review until you have resolved the above requests.

Thank you for submitting your work to PLOS One and supporting our mission of Open Science.

Response:

Thank you for bringing this issue to our attention. We have removed the previous version of S4 Dataset and uploaded a revised, fully de-identified dataset prepared in accordance with the PLOS ONE requirements for sharing human-participant data.

To minimize any potential risk of participant identification, we reduced the participant-level dataset from 44 to 10 variables and removed all variables not required to reproduce the reported Otolin-1 analyses. Specifically, we removed:

- All participant, study and hospital identification numbers, including the previously added sequential participant identifier;
- Age, sex and other demographic variables;
- Medical history and comorbidity variables;
- Blood-pressure and routine laboratory measurements;
- Exact symptom-to-sampling times, which were replaced by the prespecified analysis categories of ≤4.5 hours and >4.5–24 hours.

The revised dataset contains no names, initials, addresses, IP addresses, specific dates, contact information, hospital or study locations, original participant or hospital identifiers, or free-text information. It also contains no hidden sheets, rows or columns, comments, hyperlinks or external connections.

Only the variables necessary to reproduce the reported analyses have been retained: diagnostic classification, peripheral diagnostic category, MRI classification, plasma otolin-1 concentration, prespecified time stratum and the derived analysis-group variables. Blank cells in the derived comparison-group variables represent structural exclusions and do not contain missing identifying information. Missingness for the descriptive variables is reported only in aggregate form.

The revised file therefore contains no direct identifiers or combinations of demographic, temporal or clinical variables that could reasonably be used to identify individual participants.

The Medical Research Ethics Committee Leiden–The Hague–Delft approval for the PROVIDE study (protocol NL76143.058.21; registration number P21.029), supplied with the submission under “Other Documents,” permits the sharing of completely de-identified study data. Written informed consent was obtained from all participants. We therefore confirm that publication of the revised, fully de-identified S4 Dataset is consistent with the ethical approval, participant consent and applicable privacy requirements.

Relevant manuscript line(s): Line 497-499

6. In the online submission form, you indicated that “Data will be made available upon request and publication”
All PLOS journals now require all data underlying the findings described in their manuscript to be freely available to other researchers, either 1. In a public repository, 2. Within the manuscript itself, or 3. Uploaded as supplementary information.
This policy applies to all data except where public deposition would breach compliance with the protocol approved by your research ethics board. If your data cannot be made publicly available for ethical or legal reasons (e.g., public availability would compromise patient privacy), please explain your reasons on resubmission and your exemption request will be escalated for approval.

- The Data Availability Statement has been revised. All relevant data underlying the findings reported in this study are provided within the manuscript and its Supporting Information files. The de-identified participant-level dataset is available as S4 Dataset.

Relevant manuscript line(s): Line 497-499

7. Please upload a new copy of Figure xxxx as the detail is not clear. Please follow the link for more information:  https://journals.plos.org/plosone/s/figures

- The editorial letter does not identify a specific figure number (it states “Figure xxxx”). We have nevertheless checked the figure files against the PLOS ONE figure requirements and will upload high-resolution replacement files at resubmission. If a specific figure was intended, we would be happy to provide a further replacement.

8. Please include captions for your Supporting Information files at the end of your manuscript, and update any in-text citations to match accordingly. Please see our Supporting Information guidelines for more information: http://journals.plos.org/plosone/s/supporting-information.

- Captions for the Supporting Information figures have been added at the end of the manuscript, and the corresponding in-text citations have been standardized to S1 Fig, S2 Fig, and S3 Fig.

Relevant manuscript line(s): Supporting Information captions: lines 490-499; in-text citations: lines 251–255.

9. If the reviewer comments include a recommendation to cite specific previously published works, please review and evaluate these publications to determine whether they are relevant and should be cited. There is no requirement to cite these works unless the editor has indicated otherwise.

- No reviewer required the citation of a specific additional publication. We therefore evaluated the existing literature cited in the revised text and did not add references solely in response to a citation request.

10. Please review your reference list to ensure that it is complete and correct. If you have cited papers that have been retracted, please include the rationale for doing so in the manuscript text, or remove these references and replace them with relevant current references. Any changes to the reference list should be mentioned in the rebuttal letter that accompanies your revised manuscript. If you need to cite a retracted article, indicate the article’s retracted status in the References list and also include a citation and full reference for the retraction notice.

- We reviewed the reference list, corrected formatting and numbering inconsistencies. No cited reference required removal on the basis of retraction. The reference list has been updated throughout to remain consistent with the revised manuscript.

**Relevant manuscript line(s):** Reference list: lines 382-496

# Reviewer #1

- The manuscript is clear and well-argued. It should be of strong interest to the readership of journals focused on emergency medicine, neurology, or biomarkers.
- We thank Reviewer #1 for the positive assessment of the manuscript and for the helpful editorial suggestions. We have incorporated the suggested wording and clarifications as detailed below:
- Abstract, Methods:
  "This study used samples from a prospective multicenter cohort..." - Consider "This study analyzed samples from a prospective..."
- We agree and changed “used samples” to “analyzed samples.”

Relevant manuscript line: Line 37.

- Background: The final sentence could be rephrased slightly: "This study evaluated whether otolin-1 could serve as a diagnostic biomarker for this differentiation."
- We agree and revised the final sentence of the Abstract Background accordingly to emphasize the diagnostic-biomarker objective.

Relevant manuscript line: Line 36.

- Line 5-6: "A clinical description of symptoms that is often used, is the term acute vestibular syndrome (AVS) is acute-onset..." - This is a grammatical run-on. Suggest: "Acute vestibular syndrome (AVS) is a term used to describe acute-onset, severe dizziness..."
- We agree and corrected the sentence as suggested.

Relevant manuscript line: Line 65.

- Rationale for Otolin-1: The argument for why otolin-1 should work (inner-ear specificity) is very well laid out. However, you correctly identify its limitation (not disease-specific). The leap to "Given that central causes do not directly involve otoconial damage, it is plausible that circulating otolin-1 concentrations differ between these groups" is a testable hypothesis. You might add a sentence on the alternative hypothesis (e.g., "Conversely, if otolin-1 is a marker of chronic turnover rather than acute injury, no difference would be expected.") to strengthen the clinical equipoise
- We agree. To make the clinical equipoise explicit, we added the alternative hypothesis: “Conversely, if otolin-1 is a marker of chronic turnover rather than acute injury, no difference would be expected.”

Relevant manuscript line(s): Lines 106-107.

- Methods:
  Inclusion criteria #2: "presentation within 24 hours of symptom onset" - It's good that this is included, as it makes the hyperacute (0-4.5h) and acute (4.5-24h) subgroup analyses meaningful.
- We thank the reviewer for this observation. No change was required.
- Results, "iAOCD: isolated, acute onset, continuous dizziness": This appears to be a stray heading and can be removed from the text (it's already defined).

We thank the reviewer for this comment. The text was intended as an abbreviation definition for Table 1 rather than a separate heading. We have clarified the table note accordingly and expanded it to specify the data presentation and reference to data set and handling of data:

Relevant manuscript line(s): Lines 210-213

Abbreviations: iAOCD, isolated acute-onset continuous dizziness. Values are presented as n (%) or median [interquartile range], unless otherwise indicated. Values are based on available observations. Missing data were not imputed; variable-level missingness is reported in S4 Dataset.

- Discussion
  Paragraph 2, Line 2: "concentrations have been demonstrated in BPPV compared with healthy controls" - This is a bit repetitive of the previous sentence. Could merge or streamline.

We agree and streamlined this passage to avoid repetition. The revised text now states that otolin-1 has been proposed as a biomarker of peripheral vestibular pathology, particularly BPPV, before emphasizing that its ability to differentiate central from peripheral causes has not previously been evaluated.

Relevant manuscript line(s): Lines 281-284

- Discussion, Paragraph 4, Line 1: "An important strength of this study is the rigor of the diagnostic reference standard against which this biomarker was tested." - Excellent sentence. Keep it.

We thank the reviewer and retained this sentence unchanged.

# Reviewer #2

- General Comment: The idea of trying to identify a single peripheral tissue-derived biomarker that might be correlated with peripheral causes (in general) versus central causes of iAOCD, seems devoid of a rigorous scientific framework. The most plausible prediction would have been the actual outcome of the study: no significant difference in otolin-1 concentrations.

We appreciate this important conceptual point. We have strengthened the manuscript to make the competing biological hypotheses more explicit. In the Introduction, we now state that if otolin-1 primarily reflects chronic otolithic turnover rather than acute injury, no difference between central and peripheral groups would be expected. In the Discussion, we expanded the explanation that peripheral vestibular disorders are mechanistically heterogeneous and do not uniformly involve direct otolithic injury. These revisions better frame the study as a test of whether the previously reported peripheral-disease signal of otolin-1 generalizes to the clinically relevant central-versus-peripheral diagnostic problem.

Relevant manuscript line(s): Alternative hypothesis: lines 106-107; expanded biological interpretation: lines 306-313.

- Discussion: One could be more definitive about and expand on the following. In the context of “peripheral vestibular disorders … are biologically heterogeneous”, specifically consider some peripheral causes that do not (or might not) directly involve otoconial damage: vestibular neuritis, MS involvement of the vestibular nerve, semicircular canal dehiscence. In that context, one would not (necessarily) predict any correlation with otolin-1 concentrations.

We agree. We expanded the Discussion to clarify that peripheral vestibular disorders do not uniformly involve direct injury to otolithic structures. The revised text contrasts BPPV, which is closely related to otoconia degeneration and displacement, with vestibular neuritis, which primarily involves the vestibular nerve, and notes that disorders such as semicircular canal dehiscence may produce vestibular symptoms without primary otoconial injury. We therefore note that grouping mechanistically distinct peripheral disorders may attenuate an otolin-1 signal that is present only in specific inner-ear pathologies. We did not specifically add multiple sclerosis because its vestibular manifestations may involve central pathways and it was not a peripheral diagnostic subgroup in our cohort.

Relevant manuscript line(s): Lines 306-313.

- In Figure 2, comment/discuss on the 5 data points for the peripheral cases that are outside the y-axis (vertical) range for the central cases.

We thank the reviewer for highlighting these observations. We re-examined the five peripheral cases with otolin-1 concentrations exceeding the range observed in the central group and confirmed that these values were valid measurements rather than data-entry errors. Although these observations represent the upper tail of the peripheral distribution, there was substantial overlap between the two diagnostic groups overall, and these high values did not translate into clinically useful discrimination, as reflected by the non-significant group comparison and poor ROC performance. We added a brief descriptive statement to the Results.

Relevant manuscript line(s): Lines 219-221.

- For Figures 2, 3, 4 describe the x-axis and the distribution of data points along the x-axis. Is the variable along the x-axis continuous or discrete/categorical? If discrete/categorical, then different symbols should be used.

We thank the reviewer for this comment. The x-axis represents a categorical variable (diagnostic group: peripheral versus central). The horizontal spread of individual observations is due solely to jitter added to reduce overplotting and has no quantitative meaning. Because the categories are already explicitly separated and labelled on the x-axis, we retained the current point-symbol presentation to avoid implying that symbol shape represents an additional variable. We have clarified this point in the response rather than adding repetitive wording to each of the three figure legends.
