# Supplementary material for: Otolin-1 for differentiating central and peripheral causes of acute continuous dizziness
Source: PLoS One. 2026 Oct 22;21(10):e0361036. doi: 10.1371/journal.pone.0361036 (PMC13690215; doi:10.1371/journal.pone.0361036)
Supplement: Peer review history

# Peer review: Decision Letter 0

**DOI:** 10.1371/journal.pone.0361036.r001

**Authors:** Gauri Mankekar

6 Sep 2026

PONE-D-26-36518Otolin-1 for differentiating central and peripheral causes of acute continuous dizzinessPLOS One

Dear Dr.  Klokman,

Thank you for submitting your manuscript to PLOS One. After careful consideration, we feel that it has merit but does not fully meet PLOS One’s publication criteria as it currently stands. Therefore, we invite you to submit a revised version of the manuscript that addresses the points raised during the review process.

Please submit your revised manuscript by Oct 06 2026 11:59PM. If you will need more time than this to complete your revisions, please reply to this message or contact the journal office at plosone@plos.org. When you're ready to submit your revision, log on to https://www.editorialmanager.com/pone/ and select the 'Submissions Needing Revision' folder to locate your manuscript file.

Please include the following items when submitting your revised manuscript:

- A letter that responds to each point raised by the academic editor and reviewer(s). You should upload this letter as a separate file labeled 'Response to Reviewers'.
- A marked-up copy of your manuscript that highlights changes made to the original version. You should upload this as a separate file labeled 'Revised Manuscript with Track Changes'.
- An unmarked version of your revised paper without tracked changes. You should upload this as a separate file labeled 'Manuscript'.

If you would like to make changes to your financial disclosure, please include your updated statement in your cover letter. Guidelines for resubmitting your figure files are available below the reviewer comments at the end of this letter.

If applicable, we recommend that you deposit your laboratory protocols in protocols.io to enhance the reproducibility of your results. Protocols.io assigns your protocol its own identifier (DOI) so that it can be cited independently in the future. For instructions see: https://journals.plos.org/plosone/s/submission-guidelines#loc-laboratory-protocols. Additionally, PLOS One offers an option for publishing peer-reviewed Lab Protocol articles, which describe protocols hosted on protocols.io. Read more information on sharing protocols at https://plos.org/protocols?utm_medium=editorial-email&utm_source=authorletters&utm_campaign=protocols.

As the corresponding author, your ORCID iD is verified in the submission system and will appear in the published article. PLOS supports the use of ORCID, and we encourage all coauthors to register for an ORCID iD and use it as well. Please encourage your coauthors to verify their ORCID iD within the submission system before final acceptance, as unverified ORCID iDs will not appear in the published article. *Only*  the individual author can complete the verification step; PLOS staff *cannot*  verify ORCID iDs on behalf of authors.

We look forward to receiving your revised manuscript.

Kind regards,

Gauri Mankekar, MD,PhD,FACS

Academic Editor

PLOS One

Journal Requirements:

When submitting your revision, we need you to address these additional requirements.

1.Please ensure that your manuscript meets PLOS ONE's style requirements, including those for file naming. The PLOS ONE style templates can be found at

https://journals.plos.org/plosone/s/file?id=wjVg/PLOSOne_formatting_sample_main_body.pdf and

https://journals.plos.org/plosone/s/file?id=ba62/PLOSOne_formatting_sample_title_authors_affiliations.pdf

2. We note that the grant information you provided in the ‘Funding Information’ and ‘Financial Disclosure’ sections do not match.

When you resubmit, please ensure that you provide the correct grant numbers for the awards you received for your study in the ‘Funding Information’ section.

3. Thank you for stating the following financial disclosure:

“Funding was received from the Jeroen Bosch Hospital Stipendium, the spoedeisendegeneeskunde onderzoeksfonds (SGO), Dutch Organization for knowledge and innovation in health, healthcare and well-being (ZonMw): 10330022010010, St. Jacobusstichting and the Stichting Researchfonds Bronovo.”

Please state what role the funders took in the study.  If the funders had no role, please state: "The funders had no role in study design, data collection and analysis, decision to publish, or preparation of the manuscript."

If this statement is not correct you must amend it as needed.

Please include this amended Role of Funder statement in your cover letter; we will change the online submission form on your behalf.

4. Please note that funding information should not appear in any section or other areas of your manuscript. We will only publish funding information present in the Funding Statement section of the online submission form. Please remove any funding-related text from the manuscript.

5. When completing the data availability statement of the submission form, you indicated that you will make your data available on acceptance. We strongly recommend all authors decide on a data sharing plan before acceptance, as the process can be lengthy and hold up publication timelines. Please note that, though access restrictions are acceptable now, your entire data will need to be made freely accessible if your manuscript is accepted for publication. This policy applies to all data except where public deposition would breach compliance with the protocol approved by your research ethics board. If you are unable to adhere to our open data policy, please kindly revise your statement to explain your reasoning and we will seek the editor's input on an exemption. Please be assured that, once you have provided your new statement, the assessment of your exemption will not hold up the peer review process.

6. In the online submission form, you indicated that “Data will be made available upon request and publication”

All PLOS journals now require all data underlying the findings described in their manuscript to be freely available to other researchers, either 1. In a public repository, 2. Within the manuscript itself, or 3. Uploaded as supplementary information.

This policy applies to all data except where public deposition would breach compliance with the protocol approved by your research ethics board. If your data cannot be made publicly available for ethical or legal reasons (e.g., public availability would compromise patient privacy), please explain your reasons on resubmission and your exemption request will be escalated for approval.

7. Please upload a new copy of Figure xxxx as the detail is not clear. Please follow the link for more information:  https://journals.plos.org/plosone/s/figures

8. Please include captions for your Supporting Information files at the end of your manuscript, and update any in-text citations to match accordingly. Please see our Supporting Information guidelines for more information: http://journals.plos.org/plosone/s/supporting-information.

9. If the reviewer comments include a recommendation to cite specific previously published works, please review and evaluate these publications to determine whether they are relevant and should be cited. There is no requirement to cite these works unless the editor has indicated otherwise.

10. Please review your reference list to ensure that it is complete and correct. If you have cited papers that have been retracted, please include the rationale for doing so in the manuscript text, or remove these references and replace them with relevant current references. Any changes to the reference list should be mentioned in the rebuttal letter that accompanies your revised manuscript. If you need to cite a retracted article, indicate the article’s retracted status in the References list and also include a citation and full reference for the retraction notice.

Additional Editor Comments:

**Please see the reviewer comments and submit your responses.**

[Note: HTML markup is below. Please do not edit.]

Reviewer's Responses to Questions

**Comments to the Author**

1. Is the manuscript technically sound, and do the data support the conclusions?

The manuscript must describe a technically sound piece of scientific research with data that supports the conclusions. Experiments must have been conducted rigorously, with appropriate controls, replication, and sample sizes. The conclusions must be drawn appropriately based on the data presented.

Reviewer #1: Yes

Reviewer #2: Yes

**********

2. Has the statistical analysis been performed appropriately and rigorously?

Reviewer #1: Yes

Reviewer #2: Yes

**********

3. Have the authors made all data underlying the findings in their manuscript fully available?

The PLOS Data policy requires authors to make all data underlying the findings described in their manuscript fully available without restriction, with rare exception (please refer to the Data Availability Statement in the manuscript PDF file). The data should be provided as part of the manuscript or its supporting information, or deposited to a public repository. For example, in addition to summary statistics, the data points behind means, medians and variance measures should be available. If there are restrictions on publicly sharing data—e.g. participant privacy or use of data from a third party—those must be specified.

Reviewer #1: Yes

Reviewer #2: Yes

**********

4. Is the manuscript presented in an intelligible fashion and written in standard English?

PLOS ONE does not copyedit accepted manuscripts, so the language in submitted articles must be clear, correct, and unambiguous. Any typographical or grammatical errors should be corrected at revision, so please note any specific errors here.

Reviewer #1: Yes

Reviewer #2: Yes

**********

5. Review Comments to the Author

Please use the space provided to explain your answers to the questions above. You may also include additional comments for the author, including concerns about dual publication, research ethics, or publication ethics. (Please upload your review as an attachment if it exceeds 20,000 characters)

Reviewer #1: The manuscript is clear and well-argued. It should be of strong interest to the readership of journals focused on emergency medicine, neurology, or biomarkers.

• Abstract, Methods:

"This study used samples from a prospective multicenter cohort..." - Consider "This study analyzed samples from a prospective..."

Background: The final sentence could be rephrased slightly: "This study evaluated whether otolin-1 could serve as a diagnostic biomarker for this differentiation."

• Introduction:

Line 5-6: "A clinical description of symptoms that is often used, is the term acute vestibular syndrome (AVS) is acute-onset..." - This is a grammatical run-on. Suggest: "Acute vestibular syndrome (AVS) is a term used to describe acute-onset, severe dizziness..."

Rationale for Otolin-1: The argument for why otolin-1 should work (inner-ear specificity) is very well laid out. However, you correctly identify its limitation (not disease-specific). The leap to "Given that central causes do not directly involve otoconial damage, it is plausible that circulating otolin-1 concentrations differ between these groups" is a testable hypothesis. You might add a sentence on the alternative hypothesis (e.g., "Conversely, if otolin-1 is a marker of chronic turnover rather than acute injury, no difference would be expected.") to strengthen the clinical equipoise

• Methods:

Inclusion criteria #2: "presentation within 24 hours of symptom onset" - It's good that this is included, as it makes the hyperacute (0-4.5h) and acute (4.5-24h) subgroup analyses meaningful.

• Results, "iAOCD: isolated, acute onset, continuous dizziness": This appears to be a stray heading and can be removed from the text (it's already defined).

• Discussion”

Paragraph 2, Line 2: "concentrations have been demonstrated in BPPV compared with healthy controls" - This is a bit repetitive of the previous sentence. Could merge or streamline.

Paragraph 4, Line 1: "An important strength of this study is the rigor of the diagnostic reference standard against which this biomarker was tested." - Excellent sentence. Keep it.

Reviewer #2: General Comment

The idea of trying to identify a single peripheral tissue-derived biomarker that might be correlated with peripheral causes (in general) versus central causes of iAOCD, seems devoid of a rigorous scientific framework. The most plausible prediction would have been the actual outcome of the study: no significant difference in otolin-1 concentrations.

Specific Comments

1) Discussion: One could be more definitive about and expand on the following. In the context of “peripheral vestibular disorders … are biologically heterogeneous”, specifically consider some peripheral causes that do not (or might not) directly involve otoconial damage: vestibular neuritis, MS involvement of the vestibular nerve, semicircular canal dehiscence. In that context, one would not (necessarily) predict any correlation with otolin-1 concentrations.

2) In Figure 2, comment/discuss on the 5 data points for the peripheral cases that are outside the y-axis (vertical) range for the central cases.

3) For Figures 2, 3, 4 describe the x-axis and the distribution of data points along the x-axis. Is the variable along the x-axis continuous or discrete/categorical? If discrete/categorical, then different symbols should be used.

**********

6. PLOS authors have the option to publish the peer review history of their article (what does this mean?). If published, this will include your full peer review and any attached files.

If you choose “no”, your identity will remain anonymous but your review may still be made public.

**Do you want your identity to be public for this peer review?**  For information about this choice, including consent withdrawal, please see our Privacy Policy.

Reviewer #1: **Yes:**  Mohammed Ahmed

Reviewer #2: **Yes:**  John H Anderson

**********

[NOTE: If reviewer comments were submitted as an attachment file, they will be attached to this email and accessible via the submission site. Please log into your account, locate the manuscript record, and check for the action link "View Attachments". If this link does not appear, there are no attachment files.]

To ensure your figures meet our technical requirements, please review our figure guidelines: https://journals.plos.org/plosone/s/figures

You may also use PLOS’s free figure tool, NAAS, to help you prepare publication quality figures: https://journals.plos.org/plosone/s/figures#loc-tools-for-figure-preparation.

NAAS will assess whether your figures meet our technical requirements by comparing each figure against our figure specifications.

# Author comment: Author response to Decision Letter 1

**DOI:** 10.1371/journal.pone.0361036.r002

3 Oct 2026

Response to Editors and Reviewers

Manuscript: “Otolin-1 for differentiating central and peripheral causes of acute continuous dizziness”

We thank the Editor and both reviewers for their careful assessment and constructive comments. We have revised the manuscript accordingly. Changes are shown in the tracked-changes version and line numbers correspond accordingly.

Editor comments

1. Please ensure that your manuscript meets PLOS ONE's style requirements, including those for file naming.

- We revised the manuscript and submission package to conform to PLOS ONE style, including manuscript formatting, figure and Supporting Information nomenclature, reference formatting, and file naming. A clean manuscript and a tracked-changes manuscript are being provided separately, and figure/Supporting Information files are named to match their in-text designations.

2. We note that the grant information you provided in the ‘Funding Information’ and ‘Financial Disclosure’ sections do not match.

- We reviewed the funding information and will ensure that the Funding Information and Financial Disclosure fields in the online submission system contain identical and correct information. The ZonMw award number is 10330022010010; the remaining named funders are listed without grant numbers where no specific award number applies.

3. Please state what role the funders took in the study. If the funders had no role, please state: "The funders had no role in study design, data collection and analysis, decision to publish, or preparation of the manuscript."

If this statement is not correct you must amend it as needed.

Please include this amended Role of Funder statement in your cover letter; we will change the online submission form on your behalf.

- We have added the requested statement to the cover letter and will ensure that the online Funding Statement reflects it.

4. Please note that funding information should not appear in any section or other areas of your manuscript. We will only publish funding information present in the Funding Statement section of the online submission form. Please remove any funding-related text from the manuscript.

- The Funding section and all funding-related text have been removed from the manuscript.

Relevant manuscript line(s): Line 375 (deleted in the tracked-changes manuscript).

5. When completing the data availability statement of the submission form, you indicated that you will make your data available on acceptance. We strongly recommend all authors decide on a data sharing plan before acceptance, as the process can be lengthy and hold up publication timelines. Please note that, though access restrictions are acceptable now, your entire data will need to be made freely accessible if your manuscript is accepted for publication. This policy applies to all data except where public deposition would breach compliance with the protocol approved by your research ethics board. If you are unable to adhere to our open data policy, please kindly revise your statement to explain your reasoning and we will seek the editor's input on an exemption. Please be assured that, once you have provided your new statement, the assessment of your exemption will not hold up the peer review process.

- We have completed the data-sharing process. The de-identified participant-level dataset underlying the study findings has been added under Supporting Information as as S4 Dataset.

Relevant manuscript line(s): Line 497-499

6. In the online submission form, you indicated that “Data will be made available upon request and publication”

All PLOS journals now require all data underlying the findings described in their manuscript to be freely available to other researchers, either 1. In a public repository, 2. Within the manuscript itself, or 3. Uploaded as supplementary information.

This policy applies to all data except where public deposition would breach compliance with the protocol approved by your research ethics board. If your data cannot be made publicly available for ethical or legal reasons (e.g., public availability would compromise patient privacy), please explain your reasons on resubmission and your exemption request will be escalated for approval.

- The Data Availability Statement has been revised. All relevant data underlying the findings reported in this study are provided within the manuscript and its Supporting Information files. The de-identified participant-level dataset is available as S4 Dataset.

Relevant manuscript line(s): Line 497-499

7. Please upload a new copy of Figure xxxx as the detail is not clear. Please follow the link for more information: https://journals.plos.org/plosone/s/figures

- The editorial letter does not identify a specific figure number (it states “Figure xxxx”). We have nevertheless checked the figure files against the PLOS ONE figure requirements and will upload high-resolution replacement files at resubmission. If a specific figure was intended, we would be happy to provide a further replacement.

8. Please include captions for your Supporting Information files at the end of your manuscript, and update any in-text citations to match accordingly. Please see our Supporting Information guidelines for more information: http://journals.plos.org/plosone/s/supporting-information.

- Captions for the Supporting Information figures have been added at the end of the manuscript, and the corresponding in-text citations have been standardized to S1 Fig, S2 Fig, and S3 Fig.

Relevant manuscript line(s): Supporting Information captions: lines 490-499; in-text citations: lines 251–255.

9. If the reviewer comments include a recommendation to cite specific previously published works, please review and evaluate these publications to determine whether they are relevant and should be cited. There is no requirement to cite these works unless the editor has indicated otherwise.

- No reviewer required the citation of a specific additional publication. We therefore evaluated the existing literature cited in the revised text and did not add references solely in response to a citation request.

10. Please review your reference list to ensure that it is complete and correct. If you have cited papers that have been retracted, please include the rationale for doing so in the manuscript text, or remove these references and replace them with relevant current references. Any changes to the reference list should be mentioned in the rebuttal letter that accompanies your revised manuscript. If you need to cite a retracted article, indicate the article’s retracted status in the References list and also include a citation and full reference for the retraction notice.

- We reviewed the reference list, corrected formatting and numbering inconsistencies. No cited reference required removal on the basis of retraction. The reference list has been updated throughout to remain consistent with the revised manuscript.

Relevant manuscript line(s): Reference list: lines 382-496

Reviewer #1

• The manuscript is clear and well-argued. It should be of strong interest to the readership of journals focused on emergency medicine, neurology, or biomarkers.

- We thank Reviewer #1 for the positive assessment of the manuscript and for the helpful editorial suggestions. We have incorporated the suggested wording and clarifications as detailed below:

• Abstract, Methods:

"This study used samples from a prospective multicenter cohort..." - Consider "This study analyzed samples from a prospective..."

- We agree and changed “used samples” to “analyzed samples.”

Relevant manuscript line: Line 37.

• Background: The final sentence could be rephrased slightly: "This study evaluated whether otolin-1 could serve as a diagnostic biomarker for this differentiation."

- We agree and revised the final sentence of the Abstract Background accordingly to emphasize the diagnostic-biomarker objective.

Relevant manuscript line: Line 36.

• Line 5-6: "A clinical description of symptoms that is often used, is the term acute vestibular syndrome (AVS) is acute-onset..." - This is a grammatical run-on. Suggest: "Acute vestibular syndrome (AVS) is a term used to describe acute-onset, severe dizziness..."

- We agree and corrected the sentence as suggested.

Relevant manuscript line: Line 65.

• Rationale for Otolin-1: The argument for why otolin-1 should work (inner-ear specificity) is very well laid out. However, you correctly identify its limitation (not disease-specific). The leap to "Given that central causes do not directly involve otoconial damage, it is plausible that circulating otolin-1 concentrations differ between these groups" is a testable hypothesis. You might add a sentence on the alternative hypothesis (e.g., "Conversely, if otolin-1 is a marker of chronic turnover rather than acute injury, no difference would be expected.") to strengthen the clinical equipoise

- We agree. To make the clinical equipoise explicit, we added the alternative hypothesis: “Conversely, if otolin-1 is a marker of chronic turnover rather than acute injury, no difference would be expected.”

Relevant manuscript line(s): Lines 106-107.

• Methods:

Inclusion criteria #2: "presentation within 24 hours of symptom onset" - It's good that this is included, as it makes the hyperacute (0-4.5h) and acute (4.5-24h) subgroup analyses meaningful.

- We thank the reviewer for this observation. No change was required.

• Results, "iAOCD: isolated, acute onset, continuous dizziness": This appears to be a stray heading and can be removed from the text (it's already defined).

We thank the reviewer for this comment. The text was intended as an abbreviation definition for Table 1 rather than a separate heading. We have clarified the table note accordingly and expanded it to specify the data presentation and reference to data set and handling of data:

Relevant manuscript line(s): Lines 210-213

Abbreviations: iAOCD, isolated acute-onset continuous dizziness. Values are presented as n (%) or median [interquartile range], unless otherwise indicated. Values are based on available observations. Missing data were not imputed; variable-level missingness is reported in S4 Dataset.

• Discussion

Paragraph 2, Line 2: "concentrations have been demonstrated in BPPV compared with healthy controls" - This is a bit repetitive of the previous sentence. Could merge or streamline.

We agree and streamlined this passage to avoid repetition. The revised text now states that otolin-1 has been proposed as a biomarker of peripheral vestibular pathology, particularly BPPV, before emphasizing that its ability to differentiate central from peripheral causes has not previously been evaluated.

Relevant manuscript line(s): Lines 281-284

• Discussion, Paragraph 4, Line 1: "An important strength of this study is the rigor of the diagnostic reference standard against which this biomarker was tested." - Excellent sentence. Keep it.

We thank the reviewer and retained this sentence unchanged.

Reviewer #2

• General Comment: The idea of trying to identify a single peripheral tissue-derived biomarker that might be correlated with peripheral causes (in general) versus central causes of iAOCD, seems devoid of a rigorous scientific framework. The most plausible prediction would have been the actual outcome of the study: no significant difference in otolin-1 concentrations.

We appreciate this important conceptual point. We have strengthened the manuscript to make the competing biological hypotheses more explicit. In the Introduction, we now state that if otolin-1 primarily reflects chronic otolithic turnover rather than acute injury, no difference between central and peripheral groups would be expected. In the Discussion, we expanded the explanation that peripheral vestibular disorders are mechanistically heterogeneous and do not uniformly involve direct otolithic injury. These revisions better frame the study as a test of whether the previously reported peripheral-disease signal of otolin-1 generalizes to the clinically relevant central-versus-peripheral diagnostic problem.

Relevant manuscript line(s): Alternative hypothesis: lines 106-107; expanded biological interpretation: lines 306-313.

• Discussion: One could be more definitive about and expand on the following. In the context of “peripheral vestibular disorders … are biologically heterogeneous”, specifically consider some peripheral causes that do not (or might not) directly involve otoconial damage: vestibular neuritis, MS involvement of the vestibular nerve, semicircular canal dehiscence. In that context, one would not (necessarily) predict any correlation with otolin-1 concentrations.

We agree. We expanded the Discussion to clarify that peripheral vestibular disorders do not uniformly involve direct injury to otolithic structures. The revised text contrasts BPPV, which is closely related to otoconia degeneration and displacement, with vestibular neuritis, which primarily involves the vestibular nerve, and notes that disorders such as semicircular canal dehiscence may produce vestibular symptoms without primary otoconial injury. We therefore note that grouping mechanistically distinct peripheral disorders may attenuate an otolin-1 signal that is present only in specific inner-ear pathologies. We did not specifically add multiple sclerosis because its vestibular manifestations may involve central pathways and it was not a peripheral diagnostic subgroup in our cohort.

Relevant manuscript line(s): Lines 306-313.

• In Figure 2, comment/discuss on the 5 data points for the peripheral cases that are outside the y-axis (vertical) range for the central cases.

We thank the reviewer for highlighting these observations. We re-examined the five peripheral cases with otolin-1 concentrations exceeding the range observed in the central group and confirmed that these values were valid measurements rather than data-entry errors. Although these observations represent the upper tail of the peripheral distribution, there was substantial overlap between the two diagnostic groups overall, and these high values did not translate into clinically useful discrimination, as reflected by the non-significant group comparison and poor ROC performance. We added a brief descriptive statement to the Results.

Relevant manuscript line(s): Lines 219-221.

• For Figures 2, 3, 4 describe the x-axis and the distribution of data points along the x-axis. Is the variable along the x-axis continuous or discrete/categorical? If discrete/categorical, then different symbols should be used.

We thank the reviewer for this comment. The x-axis represents a categorical variable (diagnostic group: peripheral versus central). The horizontal spread of individual observations is due solely to jitter added to reduce overplotting and has no quantitative meaning. Because the categories are already explicitly separated and labelled on the x-axis, we retained the current point-symbol presentation to avoid implying that symbol shape represents an additional variable. We have clarified this point in the response rather than adding repetitive wording to each of the three figure legends.

Attachment Submitted filename: Response_to_Editor_Reviewers.docx

# Editor report: Decision Letter 1

**DOI:** 10.1371/journal.pone.0361036.r003

**Authors:** Gauri Mankekar

6 Oct 2026

Otolin-1 for differentiating central and peripheral causes of acute continuous dizziness

PONE-D-26-36518R1

Dear Dr. Klokman,

We’re pleased to inform you that your manuscript has been judged scientifically suitable for publication and will be formally accepted for publication once it meets all outstanding technical requirements.

Within one week, you’ll receive an e-mail detailing the required amendments. When these have been addressed, you’ll receive a formal acceptance letter and your manuscript will be scheduled for publication.

An invoice will be generated when your article is formally accepted. Please note, if your institution has a publishing partnership with PLOS and your article meets the relevant criteria, all or part of your publication costs will be covered. Please make sure your user information is up-to-date by logging into Editorial Manager at Editorial Manager® and clicking the ‘Update My Information' link at the top of the page. For questions related to billing, please contact billing support.

If your institution or institutions have a press office, please notify them about your upcoming paper to help maximize its impact. If they’ll be preparing press materials, please inform our press team as soon as possible -- no later than 48 hours after receiving the formal acceptance. Your manuscript will remain under strict press embargo until 2 pm Eastern Time on the date of publication. For more information, please contact onepress@plos.org.

We thank the reviewers for reviewing the manuscript and for their valuable time and suggestions.

Kind regards,

Gauri Mankekar, MD,PhD,FACS

Academic Editor

PLOS One

Additional Editor Comments (optional):

Reviewers' comments:

# Editor report: Acceptance letter

**DOI:** 10.1371/journal.pone.0361036.r004

**Authors:** Gauri Mankekar

PONE-D-26-36518R1

PLOS One

Dear Dr. Klokman,

I'm pleased to inform you that your manuscript has been deemed suitable for publication in PLOS One. Congratulations! Your manuscript is now being handed over to our production team.

At this stage, our production department will prepare your paper for publication. This includes ensuring the following:

* All references, tables, and figures are properly cited

* All relevant supporting information is included in the manuscript submission,

* There are no issues that prevent the paper from being properly typeset

You will receive further instructions from the production team, including instructions on how to review your proof when it is ready. Please keep in mind that we are working through a large volume of accepted articles, so please give us a few days to review your paper and let you know the next and final steps.

Lastly, if your institution or institutions have a press office, please let them know about your upcoming paper now to help maximize its impact. If they'll be preparing press materials, please inform our press team within the next 48 hours. Your manuscript will remain under strict press embargo until 2 pm Eastern Time on the date of publication. For more information, please contact onepress@plos.org.

You will receive an invoice from PLOS for your publication fee after your manuscript has reached the completed accept phase. If you receive an email requesting payment before acceptance or for any other service, this may be a phishing scheme. Learn how to identify phishing emails and protect your accounts at https://explore.plos.org/phishing.

If we can help with anything else, please email us at customercare@plos.org.

Thank you for submitting your work to PLOS One and supporting open access.

Kind regards,

PLOS One Editorial Office Staff

on behalf of

Dr. Gauri Mankekar

Academic Editor

PLOS One
